# Case Report: A giant right atrial appendage aneurysm in a child

**DOI:** 10.3389/fcvm.2024.1384972

**Published:** 2024-09-09

**Authors:** Yulan Luo, Dou Yuan

**Affiliations:** ^1^ Department of Pediatric Intensive Care Unit, West China Hospital, Sichuan University, Chengdu, Sichuan, China; ^2^ Department of Cardiovascular Surgery, Cheng Du Shang Jin Nan Fu Hospital, West China Hospital of Sichuan University, Chengdu, Sichuan, China

**Keywords:** aneurysm, right atrial appendage, surgical resection, giant aneurysm, child

## Abstract

Right atrial appendage aneurysm is an extremely rare cardiac anomaly. With unclear etiology, there is still no standard treatment method. Clinical symptoms and complications are important indicators for surgical resection. A 2-year-old boy without obvious cardiac symptoms was diagnosed with a giant right atrial outpouching arising from the right atrial appendage by computed tomography. The right atrial outpouching measured approximately 95 × 43 mm. Due to its large size and potential impact on function of right atrium and ventricle, the aneurysm was resected in surgery. During the surgery a 105 × 55 mm noncontractile cystic structure was found locating on the right anterior side of the right atrium. No other abnormalities like intracavity thrombus were detected. The patient was discharged five days after surgery. Postoperative recovery was uneventful.

## Introduction

Atrial appendage aneurysm is a very rare cardiac disease, especially right atrial appendage aneurysm. Its etiology is yet unclear. The occurrence of right atrial appendage aneurysm was reported in different age groups (1, 2). It can be asymptomatic or associated with kinds of symptoms and complications, such as dyspnea, supraventricular arrhythmia and thromboembolic diseases (1). Though there is currently no standard treatment, surgical resection is an effective treatment for right atrial appendage aneurysm. Clinical symptoms, complications, size and growth of aneurysm are important indicators for aneurysmectomy. Herein, we report a case of a giant right atrial appendage aneurysm in a child, which is extremely rare. Considering its large size and potential impact on heart function, aneurysmectomy was performed and the patient recovered well. We write to highlight this case in a child and share our experience that surgical resection can be a reasonable and safe treatment method in such a young right atrial appendage aneurysm patient.

## Case presentation

**Figure 1:**
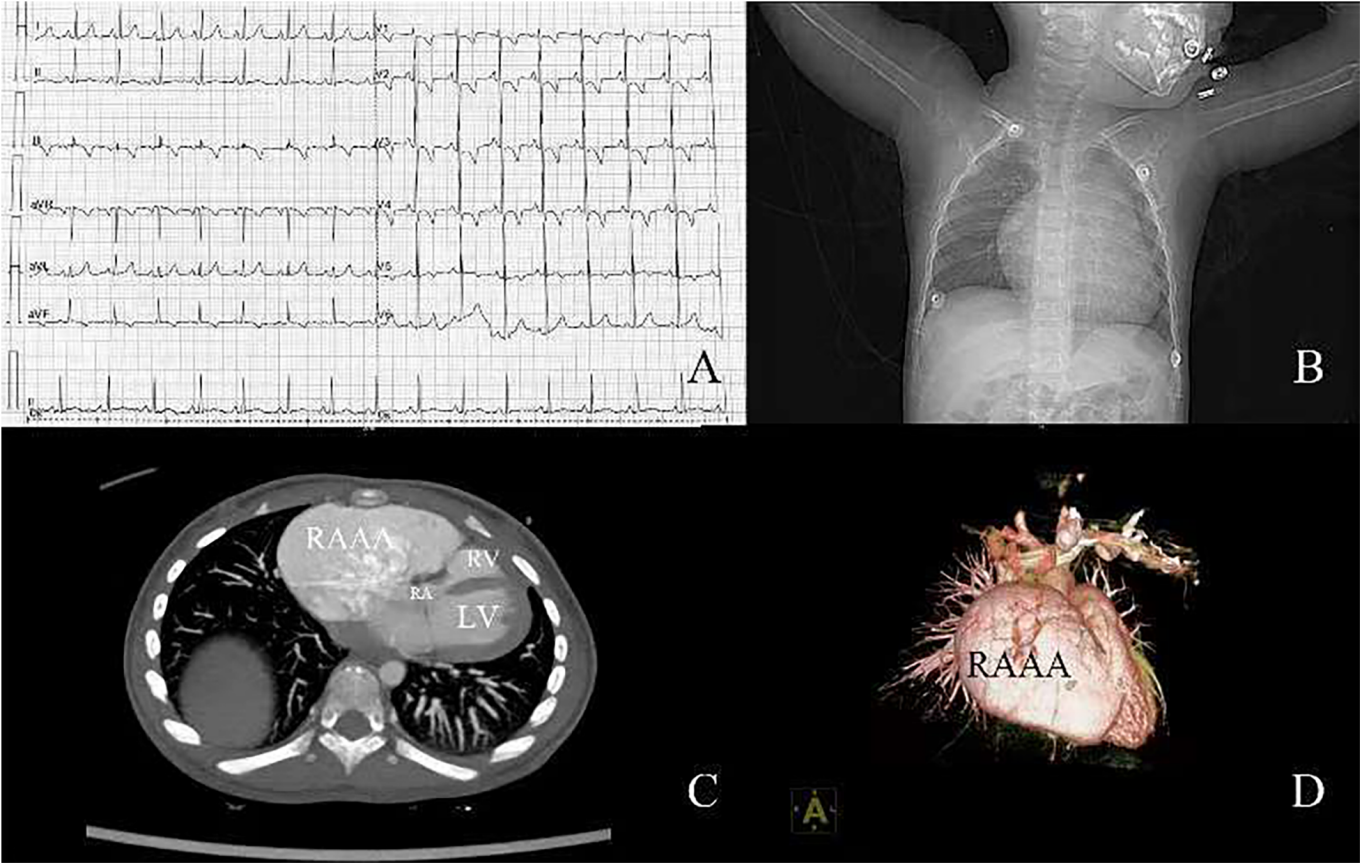
**(A)** Electrocardiogram before surgery; **(B)** chest x-ray before surgery; **(C)** contrast computed tomography; **(D)** 3D reconstruction of computed tomography. RA, right atrium; RV, right ventricle; LV, left ventricle; RAAA, right atrial appendage aneurysm.

**Figure 2:**
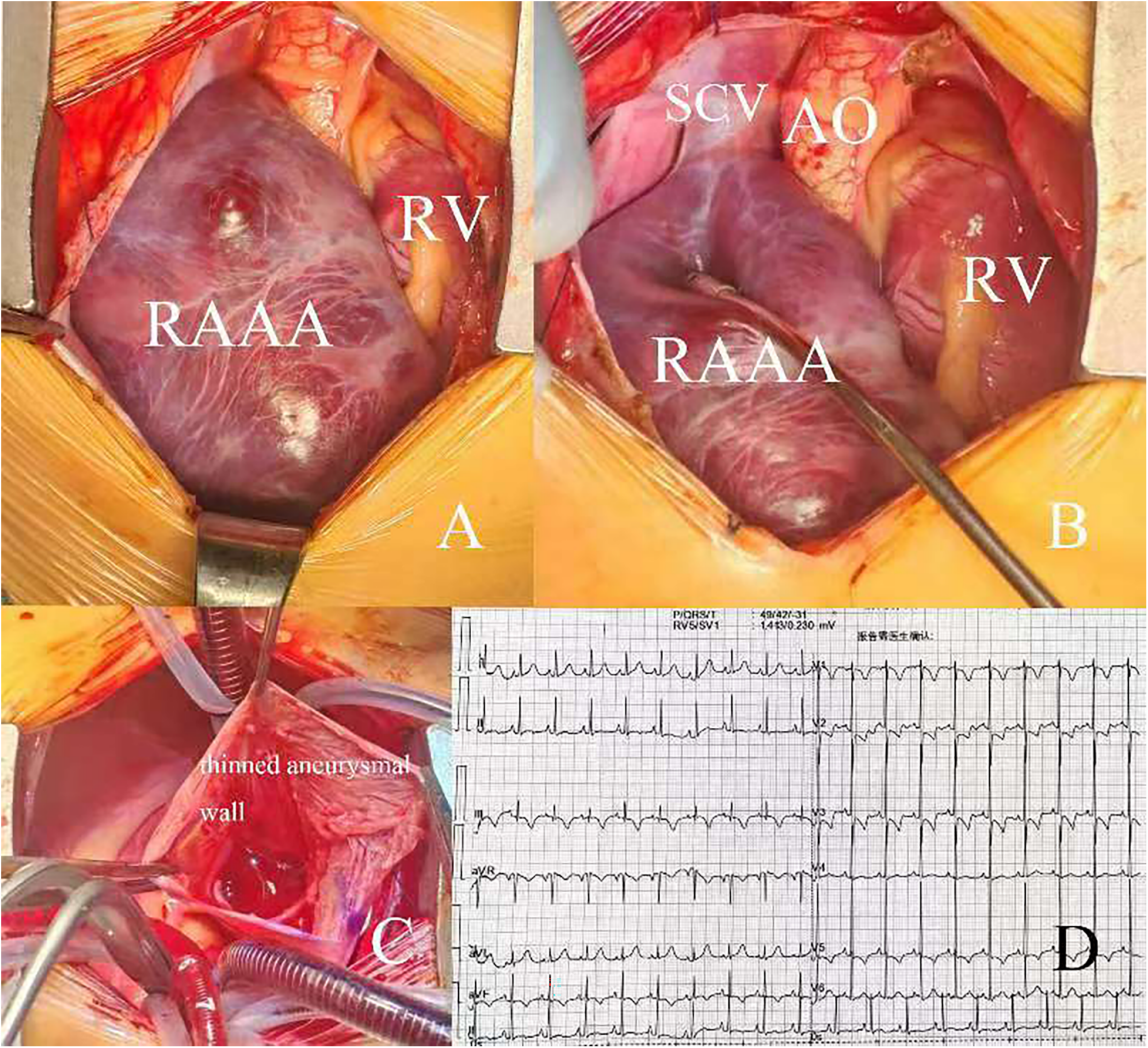
**(A,B)** intraoperative view of RAAA and its location; **(C)** intracardiac view of RAAA; **(D)** electrocardiogram after surgery. RV, right ventricle; AO, aorta; SCV, superior vena cava; RAAA, right atrial appendage aneurysm.

A 2-year-old boy was admitted to our hospital because of an enlargement of cardiac shadow incidentally revealed by chest x-ray during a routine physical examination. According to his parents, the patient had no cardiac symptoms. No heart murmur was found and the electrocardiogram showed sinus rhythm (Figure 1A). The chest x-ray showed an obvious enlargement of heart shadow (Figure 1B). Contrast computed tomography scan and 3D reconstruction demonstrated a giant right atrial outpouching arising from the right atrial appendage. It measured approximately 95 × 43 mm (Figures 1C,D). Though without clinical symptoms and complications, the patient underwent surgery to resect the aneurysm due to its large size and potential impact on function of right atrium, right ventricle and tricuspid valve caused by its compression. The operation was performed under cardiopulmonary bypass. During the surgery a 105 × 55 mm noncontractile cystic structure originating from the right atrial appendage was found locating on the right anterior side of the right atrium (Figures 2A,B). Through the incision upon the surface of the aneurysm, the inner structure of the cystic outpouching, coronary sinus and the inflows of the caval veins were checked (Figure 2C). Except for thinning wall of the aneurysm, no other abnormalities like intracavity thrombus were detected. Along the border between normal atrial wall and thinned aneurysmal wall, the aneurysm was resected. The right atrium and its connection to superior vena cava were repaired with autologous pericardium. Histopathological examination of the resected tissue from the aneurysmal wall demonstrated fibrosis of myocardium with cystic dilation and myocardial atrophy. Postoperative electrocardiogram demonstrated sinus rhythm (Figure 2D). On the fifth day after surgery, having completed all reexaminations, the patient was discharged. Postoperative recovery was uneventful.

## Discussion

The right atrial appendage aneurysm represents a rare congenital anomaly. As evidenced by literature, it manifests across various age groups, yet its occurrence in young patients, as presented in this case, is exceptionally rare (1, 2). Currently, only 6 cases of right atrial appendage aneurysm have been reported in children (3, 4). Some right atrial appendage aneurysms remain asymptomatic and may only be discovered incidentally during examinations for other conditions (5). Conversely, aneurysmal dilation of the right atrial appendage can induce symptoms such as palpitations, dyspnea, and exertional fatigue (6, 7). Furthermore, these aneurysms are frequently associated with serious complications, including supraventricular arrhythmia, intracardiac thrombus formation, right atrial systolic dysfunction, and even right ventricular dysfunction due to compression (8, 9).

Given the potential severity of complications, early and accurate diagnosis of right atrial appendage aneurysm is crucial. In addition to computed tomography (CT) and magnetic resonance imaging (MRI), echocardiography also serves as an effective and timely diagnostic tool. Compared to MRI and CT, echocardiography is particularly advantageous for disease screening due to its accessibility and efficiency.

Currently, there is no standardized treatment for right atrial appendage aneurysms. The choice between surgical resection, drug therapy, or simple follow-up depends on the clinical symptoms, complications, and the size of the aneurysm. In the absence of symptoms or complications, surgical intervention is generally deemed unnecessary. Instead, regular follow-up and anticoagulant therapy to prevent thromboembolic disease may suffice (10). However, in cases where symptoms or complications arise, or the aneurysm is large or growing, surgical resection combined with medical treatment becomes a common therapeutic strategy (11, 12). If the patient concurrently exhibits supraventricular arrhythmia resistant to antiarrhythmic medications, surgical resection alongside concomitant ablation may be considered a reasonable approach (13, 14). Nevertheless, there are exceptions. For instance, if medical treatment or electrocardioversion successfully restores sinus rhythm, and no other symptoms or complications necessitate surgical intervention, patients may opt out of surgery (15). Additionally, some patients requiring operations for other cardiac conditions might have the aneurysm removed opportunistically during the procedure. For example, during surgery for atrial and ventricular septal defects, an aneurysm resection may also be performed incidentally (16). Similarly, in cases where coronary artery bypass grafting is planned for treating anomalous coronary artery origins, the aneurysm may be resected as a matter of convenience (17). Moreover, in scenarios where transcatheter intervention is sufficient to close an atrial septal defect, surgical intervention may be deemed unnecessary, leaving the right atrial appendage aneurysm untouched (1).

In this case, given that the aneurysm was very large and could lead to compression, which might influence the function of right ventricle and tricuspid valve, it was resected. After the surgery, the patient recovered very well. As in this case, most patients with right atrial appendage aneurysms, when get appropriate treatment, can have good prognosis.

## Conclusion

As a very rare disease, right atrial appendage aneurysm is found in both child and adult patients. Concomitant symptoms and complications are key factors in determining therapeutic Modalities. With proper treatment, patients with right atrial appendage aneurysm can achieve a good prognosis.

## Data Availability

The original contributions presented in the study are included in the article/Supplementary Material, further inquiries can be directed to the corresponding author.
